# Autism Spectrum Disorders traits in a sample of young adults referring to a generalized mental health outpatient clinic

**DOI:** 10.1192/j.eurpsy.2024.1183

**Published:** 2024-08-27

**Authors:** V. Nistico’, I. Folatti, G. Santangelo, C. Sanguineti, S. Inci, R. Faggioli, A. Bertani, O. Gambini, B. Demartini

**Affiliations:** ^1^Health Science Department, University of Milan; ^2^Department of Psychology, University of Milan Bicocca; ^3^ Aldo Ravelli Research Center for Neurotechnology and Experimental Brain Therapeutics, University of Milan; ^4^UO Psichiatria 51 e 52; ^5^Centro Giovani Ettore Ponti, ASST Santi Paolo e Carlo, Presidio San Paolo, Milan, Italy

## Abstract

**Introduction:**

The diagnosis of Autism Spectrum Disorders is currently witnessing several changes, with direct consequences on the prevalence rates in the general population. However, little is known about ASD traits prevalence in clinical samples, and how much these traits interact with other mental health conditions, especially in young adults, a critical age for the outbreak of many psychiatric diseases.

**Objectives:**

The aim of this study was to assess the prevalence of ASD traits in a sample of young adults (aged between 18 and 24 years old) referring to a specialized mental health outpatient clinic.

**Methods:**

We administered to 259 patients the Autism Quotient (AQ) and the Ritvo Autism and Asperger Diagnostic Scale-Revised (RAADS‐R), along with a detailed sociodemographic and anamnestic interview.

**Results:**

We found that 16.2% of our sample scored above the cut-off at both scales (a percentage that went down to 13.13% when restricting the RAADS-R cut-off at >119, as suggested for clinical samples).

**Conclusions:**

This prevalence seems considerably higher than the one reported in the general population, and not negligible. The association with sociodemographic features such as sex assigned at birth, gender identity and employment status, and the validity of the screening tools we implemented, are discussed. In conclusion, we suggest that an assessment for autistic traits should be implemented in young adults seeking help for unspecified psychiatric symptoms and psychological suffering and that, despite the not unanimous consensus over self-report screening tools, a positivity to both the AQ and the RAADS-R should lead the clinician to conduct a full diagnostic evaluation with structured or semi-structured interviews.

**Disclosure of Interest:**

None Declared

